# Molecular Evidence of *Wolbachia* Species in Wild-Caught *Aedes albopictus* and *Aedes aegypti* Mosquitoes in Four States of Northeast India

**DOI:** 10.1155/2023/6678627

**Published:** 2023-09-05

**Authors:** Sathishkumar Vinayagam, Tulika Nirmolia, Sumi Chetry, Narendran Pradeep Kumar, Prasanta Saini, Dibya Ranjan Bhattacharyya, Ipsita Pal Bhowmick, Kamaraj Sattu, Saurav Jyoti Patgiri

**Affiliations:** ^1^ICMR-Regional Medical Research Centre North East Region, Dibrugarh, Assam 786010, India; ^2^Periyar University, Centre for PG & Research Studies, Dharmapuri 635205, India; ^3^ICMR-Vector Control Research Centre, Puducherry 605006, India

## Abstract

*Wolbachia*, a Gram-negative intracellular bacterium, naturally infects many arthropods, including mosquito vectors responsible for the spread of arboviral diseases such as Zika, chikungunya, and dengue fever. Certain *Wolbachia* strains are involved in inhibiting arbovirus replication in mosquitoes, and this phenomenon is currently being studied to combat disease vectors. A study was conducted in four states in north-eastern India to investigate the presence of natural *Wolbachia* infection in wild-caught *Aedes albopictus* and *Aedes aegypti* mosquitoes, the established vectors of dengue. The detection of a *Wolbachia* infection was confirmed by nested PCR and sequencing in the two mosquito species *Ae. aegypti* and *Ae. albopictus*. Positivity rates observed in *Ae. aegypti* and *Ae. albopictus* pools were 38% (44 of 115) and 85% (41 of 48), respectively, and the difference was significant (chi-square = 28.3174, *p* = 0.00000010). Sequencing revealed that all detected *Wolbachia* strains belonged to supergroup B. Although *Wolbachia* infection in *Ae. aegypti* has been previously reported from India, no such reports are available from north-eastern India. Data on naturally occurring *Wolbachia* strains are essential for selecting the optimal strain for the development of *Wolbachia*-based control measures. This information will be helpful for the future application of *Wolbachia*-based vector control measures in this part of the country.

## 1. Introduction

Vector-borne diseases cause significant loss of life in terms of morbidity and mortality. In north-eastern (NE) India, dengue fever and malaria are endemic and outbreaks are common in various states in the region [1–4]. Mosquito vectors such as *Ae. aegypti* and *Ae. albopictus* cause many dengue outbreaks each year and traditional measures to combat them have not yielded the expected results [5]. Increasing insecticide resistance among the mosquito population further exacerbates this problem. In India, both mosquito species showed different degrees of resistance to dichlorodiphenyltrichloroethane (DTT) in most states [6, 7]. Therefore, more effective and biologically active vector control measures are needed to prevent these vector-borne diseases [8]. *Wolbachia*, a class of Alphaproteobacteria, is endosymbiotic in several arthropods and filarial nematodes in the biosphere. There are currently 17 supergroups of endosymbiotic *Wolbachia* (A–S, excluding G and R), most of which infect terrestrial arthropods, particularly insects and arachnids [9–11]. Interestingly, certain *Wolbachia* species have been shown to possess a natural ability to alter the biology of the infected host mosquito, making them less susceptible to infection by arboviruses such as dengue virus (DENV), chikungunya virus (CHIKV), yellow fever virus (YFV), and Zika virus (ZIKV). This property has been exploited by various groups to transfect *Ae. aegypti* with *Wolbachia* strains such as *w*Mel, wMelPop − CLA, *w*AlbB, and *w*Mel/*w*AlbB [5]. In Orissa, genetically distinct and unique *Wolbachia* species have been reported in the coastal plains, which show completely different characteristics from the other populations of the country [12]. In addition, both *w*AlbA and *w*AlbB*Wolbachia* endosymbionts were observed in *Ae. albopictus* population from the Andaman and Nicobar Islands [13]. A low prevalence of these *Wolbachia* endosymbionts has been observed in Indian wild mosquitoes of *An. culicifacies* and *An. stephensi* species from Tamil Nadu [14]. However, there can be significant overlap in *Wolbachia* strains infecting one host, and different strains can affect the survival of the other [15]. Geographical and ecological factors must also be taken into account. This could have an important impact on the selection of the optimal strain for transfection in *Wolbachia*-based vector control strategies since a detailed assessment of native strains in mosquito populations is first required. The current study was conducted to detect *Wolbachia* infection in adult *Ae. aegypti* and *Ae. albopictus* mosquitoes collected in four different states (Assam, Arunachal Pradesh, Nagaland, and Meghalaya) in NE India by nested PCR using 16S rRNA-specific primers followed by sequencing.

## 2. Materials and Methods

**Figure 1:**
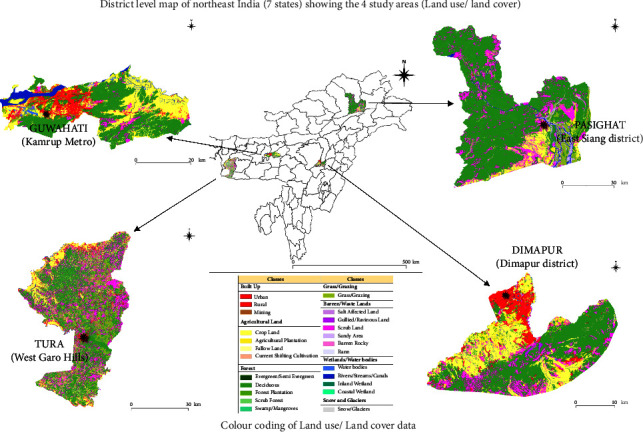
District-level map of NE India (7 states) showing the four study areas (GPS coordinates marked with black ^∗^sign) with land use/land cover details. (Data source: IRS P6 LISS 111 satellite data from National Remote Sensing Centre, Indian Space Research Organization, Department of Space, Govt. of India, Balanagar, Hyderabad-500037; image created with Bhuvan (https://bhuvan.nrsc.gov.in/) and QGIS version. 3.8.3-Zanzibar).

We used archived mosquito samples collected as part of the previously conducted project “Vector Surveillance for ZIKV in Selected High-Risk Areas” [16, 17]. *Ae. albopictus* and *Ae. aegypti* mosquitoes (adult) were sampled from February 2018 to February 2019 in urban areas from four dengue-prone regions in four different states in NE India: Guwahati (Kamrup Metro district, Assam), Tura (West Garo Hills district, Meghalaya), Pasighat (East Siang district, Arunachal Pradesh), and Dimapur (Dimapur district, Nagaland). The location of the study sites is shown in Figure 1. Adult mosquitoes were originally collected using suction tubes from indoor and outdoor resting sites such as open water tanks, garages, tire dumps, and leaf axils. Mosquitoes collected in the field were separated according to species, collection site, date, sex, and blood-fed status of the female mosquitoes. A maximum of 20 mosquitoes were pooled in one tube and transported to the laboratory in 50 *μ*L of TRI Reagent (Molecular Research Center, Inc., USA) at 4°C. Samples were uniformly homogenized, and DNA was extracted using the commercially available DNeasy Blood and Tissue Kit (Qiagen) and stored at −20°C until further processing.

With some minor modifications, ITS2-PCR (Internal Transcribed Spacer-2) was performed to validate *Ae. aegypti* and *Ae. albopictus* species in the collected mosquito pools [18]. The PCR contained 1.0 mM MgCl_2_ and the primers ITS2-F and ITS2-R (5′-ATCACTCGGCTCGTGGATCG-3′,5′-ATGCTTAAATTTAGGGGGTAGT-3′) at a concentration of 1 *μ*M each. The PCR settings were as follows: 95°C for 5 minutes (initial denaturation), then 35 cycles of 95°C for 30 seconds (denaturation), 56°C for 30 seconds (annealing), 72°C for 45 seconds (extension), and 72°C for ten minutes the last extension. Positive controls for *Ae. albopictus* were provided by the Indian Council of Medical Research-Vector Control Research Center (ICMR-VCRC, Puducherry) and internal controls were used for *Ae. aegypti*. *Wolbachia* detection in *Aedes* mosquitoes was performed using nested PCR (nPCR) as described by Shaw et al. [19]. From the extracted individual pools of *Ae. aegypti* and *Ae. albopictus* gDNA, 16S rRNA *Wolbachia* gene was targeted by nested PCR. The initial PCR was performed with *Wolbachia* 16S rRNA-specific primer pairs (W-Specf: 5-CATACCTATTCGAAGGGATAG-3 and W-Specr: 5-AGCTTCGAGTGAAACCAATTC-3) in a 25 *μ*L reaction volume with 5 *μ*L of gDNA. Then, 5 *μ*L of the primary PCR amplicon was used as a target in the second round of PCR with the following internal primers: 16SNF (5-GAAGGGATAGGGTCGGTTCG-3) and 16SNR (5-CAATTCCCATGGCGGTGACG-3) in a reaction volume of 50 *μ*L. The PCR protocol for nested 16S rRNA PCR was as follows: initial denaturation at 95°C for 15 minutes, followed by 35 cycles of 15 seconds at 95°C, 25 seconds at 66°C, and 30 seconds at 72°C; followed by a final extension step at 72°C for 5 minutes [19]. *Wolbachia* control DNA provided by ICMR-VCRC, Puducherry, was used as a PCR positive control, and double distilled water (ddH_2_O) was used as a negative control. The secondary PCR product, 412 bp in size, was considered specific for *Wolbachia* and sequenced using Sanger's technique. The *Wolbachia* 16S and *Aedes* ITS2 sequences obtained were checked for sequence quality and compared using the Bioedit Version 7.2 software [20]. The aligned nucleotide sequences were checked for matches and compared to pre-existing high-similarity sequences downloaded from the NCBI GenBank database. All sequences were aligned with Clustal W and exported to MEGA X software for further genetic analysis [21].

## 3. Results

**Figure 2:**
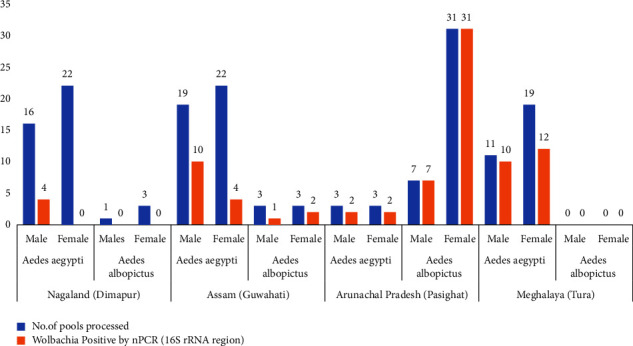
Frequency distribution and characteristics of *Wolbachia* positive pools by nPCR.

**Figure 3:**
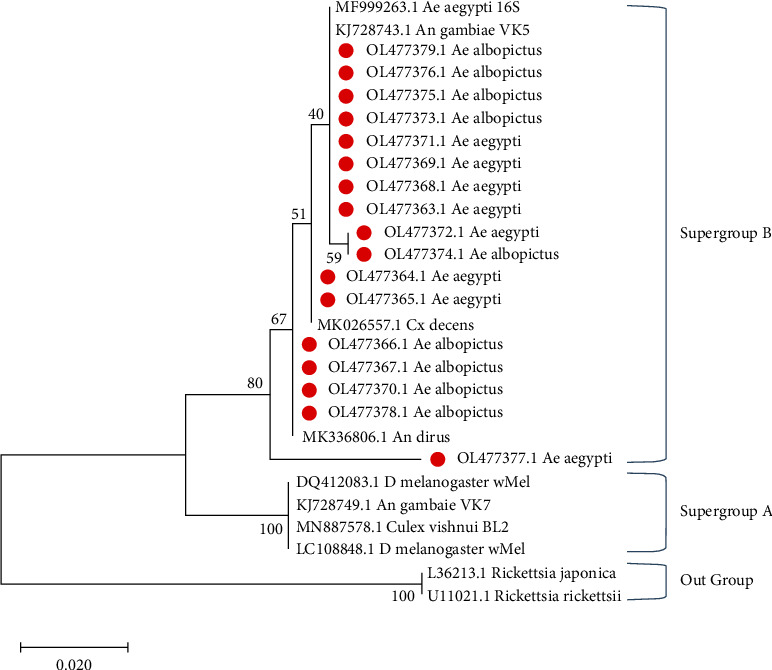
Phylogenetic analysis of *Wolbachia* strains isolated in the study (maximum likelihood method). Red dots indicate samples from the current study.

**Figure 4:**
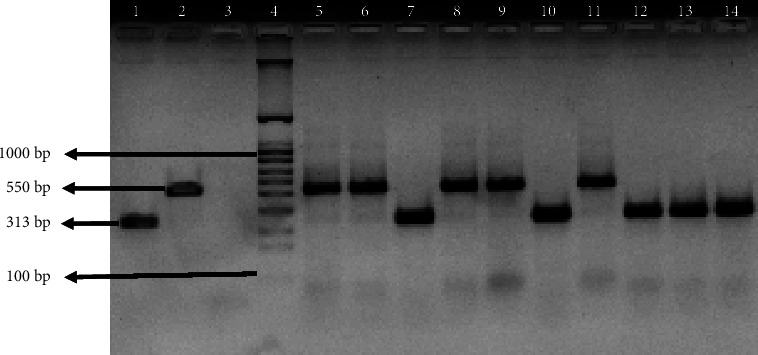
PCR amplified ITS2 nuclear gene fragments with respective controls of *Ae. aegypti* and *Ae. albopictus* (lanes 1 and 2 are positive controls of *Ae. aegypti* and *Ae. albopictus*, lane 3: negative control, lane 4:1 kb marker, and lanes 5–14 are sample pools of different states of northeast India).

**Figure 5:**
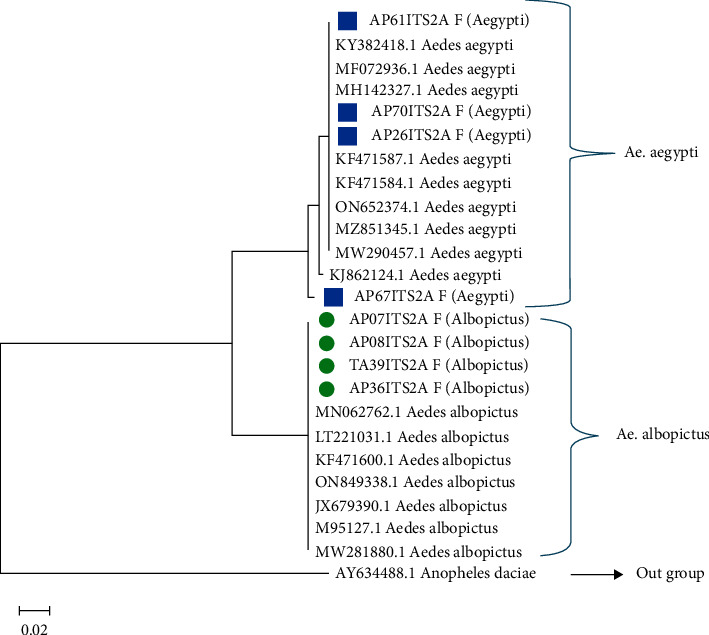
Neighbour-joining tree showing the taxonomic characterization of *Ae. aegypti* and *Ae. albopictus ***(**blue and green denotes isolates from the current study**)**.

The project collected a total of 6,229 adult *Aedes* mosquitoes from dengue-endemic areas in four different Northeast states. Details on the distribution of these mosquitoes can be found elsewhere [17]. In short, it was found that *Ae. aegypti* was the predominant *Aedes* species (63.3%) among all mosquitoes collected in the study. In Guwahati, Dimapur, and Tura, which are predominantly urban areas, *Ae. aegypti* was dominant, while in Pasighat, which is surrounded by forested areas, *Ae. albopictus* was predominant [17]. From a total of 515 pools, 163 representative pools from the four regions were randomly selected for the current study. Since *Wolbachia* infection in *Ae. albopictus* is already established in this region, a larger number of *Ae. aegypti* pools were selected for analysis (115 vs. 48). Of the 163 pools, a total of 85 pools were found positive for *Wolbachia* by nPCR (Table 1, Figure 2). Positivity rates observed in *Ae. aegypti* and *Ae. albopictus* were 38% (44 of 115) and 85% (41 of 48), respectively, and the difference was significant (chi-square = 28.3174, *p* = 0.00000010). A total of 17 *Wolbachia* 16S PCR amplicons (8 from *Ae. aegypti* and 9 from *Ae. albopictus* pools) were sequenced in the current study using Sanger's technique. The resulting sequences have been deposited in the NCBI GenBank database (accession numbers: OL477363.1-OL477379.1). The phylogenetic maximum likelihood tree was constructed using the Tamura–Nei model [22]. The tree with the highest log likelihood (−663.46) comprised 27 nucleotide sequences (295 positions each) (Figure 3). MEGA X was used to perform evolutionary analysis [21]. When compared to other known sequences from groups A and B, it was found that all isolates in the current study belonged to *Wolbachia* supergroup B. For the confirmation of the field-collected *Aedes* samples as *Ae. aegypti* and *Ae. albopictus*, an ITS2-PCR was performed with corresponding positive controls (Figure 4). A total of 8 ITS2-PCR amplicons (4 *Ae. aegypti* and 4 *Ae. albopictus*) were sequenced and submitted to the NCBI GenBank (ITS2 sequence accession numbers: OP327745 – OP327752). A total of 25 nucleotide sequences were used to construct the phylogenetic tree using the neighbour joining method and the Kimura-2 parameter in MEGA 11 [23, 24]. Analysis of the phylogenetic tree (Figure 5) showed good agreement of the study samples with *Ae. aegypti* and *Ae. albopictus* [18, 25].

## 4. Discussion

The current study has shown the prevalence of *Wolbachia* species in two important dengue vectors viz. *Ae. aegypti* and *Ae. albopictus* from four dengue endemic areas spread over four states in northeastern India. In both mosquito species, *Wolbachia* supergroup B was detected. The positivity rate was higher in *Ae. albopictus* compared to that of *Ae. aegypti* (85% vs. 38%), and the difference was significant. Although previous studies from Northeast India have reported *Wolbachia* in various mosquito vectors, there are no previous reports of *Wolbachia* infection in *Ae. aegypti* [15]. Traditionally, it was assumed that natural infection of *Ae. aegypti* with *Wolbachia* was not common [15, 26]. Previously, researchers used different sets of primers to detect natural infection of *Wolbachia* in mosquito vectors. *Wolbachia* surface protein (*wsp*)-based primers have been widely used to detect *Wolbachia* superinfections in many arthropods. A comparison between *wsp* primers and 16SrRNA-based primers in *Ae. aegypti* mosquitoes has shown that the highest detection rate was achieved with 16SrRNA primers in the US [27]. Malaria vectors such as Anopheles mosquitoes were also assumed not to be naturally infected with *Wolbachia* until a unique 16SrRNA-based PCR demonstrated that *An. gambiae* carry low-level natural *Wolbachia* infection [19].

Subsequently, researchers from Malaysia (25%), the Philippines (16.8%), and the USA (44.8%) have reported natural *Wolbachia* infection in *Ae. aegypti* using *Wolbachia* 16SrRNA primers [27–29]. In 2019, the natural infection of *Ae. aegypti* with *Wolbachia* supergroup B was detected using 16SrRNA-based primers from Coimbatore, Tamil Nadu, India [5]. The reported strain showed 99% homology with the *w*AlbB strain in *Ae. albopictus* [5]. This is similar to the homology levels observed in our study (98%–99%) compared to published sequences in the NCBI database. The abovementioned studies from Malaysia, Philippines, USA, and India observed *Wolbachia* infection in *Ae. aegypti* mosquitoes by screening individual mosquitoes. However, *Wolbachia* infection in screened pools of *Ae. aegypti* has also been described previously [30, 31]. Coon et al. reported two *Wolbachia* 16S rDNA OTUs (operational taxonomic units) in a pool of 30 *Ae. aegypti* larvae collected in Florida in 2014 [30]. Similarly, *Wolbachia* 16S OTUs have been detected in pooled *Ae. aegypti* mosquitoes in Thailand [31].

In most countries, including India, *Ae. albopictus* mosquitoes have traditionally been subjected to *Wolbachia* detection using primers based on *Wolbachia* surface protein (*wsp*). In a study conducted in Orissa, India, 1291 male and female *Ae. albopictus* mosquitoes collected from 15 districts across the state were tested for *Wolbachia* infection using *wsp* primer-based PCR. Among these, 1281 (99.2%) mosquitoes tested positive for *Wolbachia* infection; most were individually infected with supergroups B and A, and some had mixed infection with A and B [12]. Another study from Orissa found that 5% of *Ae. albopictus* were monoinfected with *w*AlbA, 10% with *w*AlbB and 80% with both *w*AlbA and *w*AlbB [32]. Similarly, Ravikumar H et al. also reported *Wolbachia* infection in eight of twenty mosquito species using *wsp* primer sets, with *Wolbachia* A and B observed in *Ae. albopictus* mosquitoes [33]. From Assam, NE India, Soni et al. showed superinfection with *Wolbachia* A and B from Dibrugarh, Tinsukia, and Sibsagar districts; whereas from Tezpur, in *Ae. albopictus*, only the *Wolbachia* supergroup A was detected. [15]. In the Andaman and Nicobar Islands, a total of 57 *Ae. albopictus* mosquitoes (100%) were found to be infected with *Wolbachia* supergroups A and B by *wsp* primer-based PCR [13].

The *w*AlbB strain has been shown to confer protection against arboviruses in mosquito vectors. However, the route of infection seems to play a crucial role. Whether a particular mosquito species is infected with this strain naturally, through transient or stable transfection, has a major impact on resistance to arboviruses such as dengue and chikungunya [34, 35]. While natural infection of *Ae. albopictus* with *w*Alb A and *w*AlbB showed no antiviral activity, stable transinfection with the wMel strain has been reported to reduce transmission of DENV and CHIKV [34, 35]. Likewise in the case of *Ae. aegypti*, stable transinfection with wMelPop, *w*AlbB, and wMel strains all showed reduced infection rate, viral load, and transmission rates for DENV and CHIKV compared to *Wolbachia*-free mosquitoes [34, 36]. However, such studies on natural populations are scarce and large-scale studies across different geographic locations are needed to obtain conclusive evidence for the potential antiviral role of wild-caught, naturally infected *Ae. aegypti* and *Ae. albopictus*. Different types of *Wolbachia* strains provide different survival benefits to host mosquitoes and may also entail fitness costs. One variety can also complement or compete with the other. Experimenting with *Ae. albopictus* triple infections with wMel, *w*AlbA, and *w*AlbB has shown that the introduction of new *Wolbachia* strains can sometimes lead to unexpected complications in uninfected or naturally infected mosquito vectors [36–38]. Our observation on the molecular detection of *Wolbachia* using 16SrRNA primer-based PCR and sequencing in *Ae. aegypti* and *Ae. albopictus* could have important implications for future intervention strategies based on the transfection of *Wolbachia* strains on *Aedes* mosquitoes. It may or may not play an important role in reducing arbovirus transmission under natural conditions. However, further validation of the definitive presence of natural infection in these mosquito hosts requires additional molecular tests such as the detection of bacteria in the host tissue and their removal after antibiotic treatment and whole genome sequencing [39]. We propose that natural infection of *Wolbachia* in mosquito vectors needs to be delineated, preferably based on multiple lines of evidence in different geographic regions, before initiating vector control measures based on *Wolbachia*-infected mosquitoes.

## Figures and Tables

**Table 1:** Distribution and characteristics of *Wolbachia* positive pools by nPCR.

Locations	Mosquitoes species	Gender	No. of pools processed	*Wolbachia* positive pools by nPCR	Pool positivity rate (%)
Nagaland (Dimapur)	*Aedes aegypti*	Male	16	4	25.00
		Female	22	0	0
	*Aedes albopictus*	Males	1	0	0
		Female	3	0	0
		Total	42	4	9.52

Assam (Guwahati)	*Aedes aegypti*	Male	19	10	52.60
		Female	22	4	18.10
	*Aedes albopictus*	Male	3	1	33.30
		Female	3	2	66.60
		Total	47	17	36.17

Arunachal Pradesh (Pasighat)	*Aedes aegypti*	Male	3	2	66.60
		Female	3	2	66.60
	*Aedes albopictus*	Male	7	7	100
		Female	31	31	100
		Total	44	42	95.45

Meghalaya (Tura)	*Aedes aegypti*	Male	11	10	90
		Female	19	12	63.10
	*Aedes albopictus*	Male	0	0	0
		Female	0	0	0
		Total	30	22	73.33

## Data Availability

All the sequences generated in this study have been submitted to the NCBI GenBank and can be obtained from the database. The relevant Accession Numbers are mentioned in the text.
